# Topics of Public Interest

**Published:** 1915-10

**Authors:** 


### TOPICS OF PUBLIC INTEREST.

---

**Statistics of Medical Education.** (J. A. M. A., August 21, 1915). The total number of medical graduates for 1915 was 3,536 the smallest number since 1890 and only slightly greater than in 1880. The maximum number of graduates was 5,747 in 1904. The diminution in graduates from 1914 to 1915 was only 58 but the number of medical students decreased from 16,502 to 14,891 so that there must be a considerable further decrease for the next three years, at least. A favorable economic factor noted for several years is that, in the aggregate, the senior classes of medical schools have shown a steady decrease in numbers, in other words that there have been only slight fluctuations in matriculation and that, generally speaking, the number of graduates has been close to a quarter of the total number of students, indicating a slight economic loss in taking partial courses. Sectarian graduates are now confined to a few homoeopathic and eclectic schools, number about 7% of the total and do not present any real problem in medical solidarity. Women graduates are relatively fewer than several years ago, averaging less than 4% of the total.

Preliminary education is now on a highly satisfactory basis. The percentage of graduates holding bachelor's degrees has gradually increased from 15.3% in 1910 to 24.3% in 1915 and is, indeed, 25.5% for graduates of "regular schools." The number of medical schools has decreased, mainly by mergers, from the maximum of 162 in 1906 to 95 in 1915. Of these 83 are regular, 8 homoeopathic and 4 eclectic. 83 of the 95 medical schools require at least one year of college study beyond a full high school course and 39 require two years. Of the 39, 7 will require 2 years college work in a year or two. 66 colleges are classified as in Class A; 17 in Class B; 12 in Class C. 74.4% of the graduates of 1915 were from Class A colleges, 19.4% from Class B, 6.2% from Class C.

---

**The Hospital of the Good Shepherd,** Syracuse, will be used for clinical teaching in connection with Syracuse University.

---

**Influence of War on Births by Sex.** There has long been a belief that, in some unknown way, war tended to compensate for the preponderance of male deaths by inducing a preponderance of male births. Henri Coupin, Presse Med. July 26, confirms this idea by the following statistics: Of 559 children born to fugitives from Galicia and Bukovina, 314 were boys. In Vienna, since October 1914, the sex birth rate has changed from 108 males: 100 females to 140:100.

---

**Typhoid in Cincinnati, 1914.**

| | Cases. | Deaths. |
|---|---|---|
| Imported | 28 | 7 |
| Away from city within 30 days prior to illness | 36 | 4 |
| Drank well and cistern water | 8 | 1 |
| Drank water directly from river, springs, etc. | 16 | 1 |
| Drank turbid water following break in city main | 4 | 0 |
| Diagnosis doubtful | 4 | 3 |
| Source of infection unknown | 45 | 7 |
| Total | 148 | 23 |

---

**Weather Statistics.** There has been a general impression that the past summer has been marked by an unusual amount of rain. Judging from the condition of the roads and fields and the complaints by farmers of ruined crops, especially of grain which could not be cut and mowed away until it was dead ripe and darkened, and of potaoes rotted in the ground, this impression seemed correct. The U. S. Weather Bureau, Buffalo station, however, reports as follows: May, 13 days rain, (0.01 inch or more on a day); June, 11 days; July, 16 days; August, 12 days. The total rain fall by months was: 1.86 inches, 1.72, 3.37, and 6.19; total for four months 13.14 inches, about half an inch more than the average. The August total was nearly double the average but, on account of an unusually dry April, the total rain fall for the first eight months of the year 1915 is slightly below the average. The medical profession is well aware of the danger of trusting to general impressions, although humanly prone to be led astray by them. It is worth while to inquire however, whether the genuine excess of rain during August, has had any measurable influence on disease incidence, either on account of exposure or by overflow of ordinary drainage channels during the vacation season.

---

**Interne Wanted** at St. Luke's Hospital, Utica. Inquire at this office or of our associate editor, Dr. Willis E. Ford of Utica.

---

**Medical Inspection of Schools at North Tonawanda.** Dr. F. W. Bentley's report for the last school year included 2,050 examinations. 426 showed defective vision, 367 defective hearing, 114 enlarged glands, 214 enlarged tonsils, 278 bad teeth.

---

**The Buffalo Homoeopathic Hospital** has received $9,500 by bequest of the late Dr. Edward Gregg of Pittsburgh, $500 having been deducted for the Pennsylvania state tax. An inheritance tax on any reasonable sum, amounting to a year's income from the principal is of questionable propriety and certainly, money left to charitable institutions of any kind should not be taxed at all.

---

**Fire in the Garage of the Buffalo General Hospital,** early in the morning of September 7, destroyed one ambulance and badly damaged another.

---

**New York State Vital Statistics** for the first half of 1915, as compared with similar statistics for 1915 and the average for 1910-1914, show slight improvement all along the line, except for cancer, which has increased to 89.8 deaths per 1,000 population, as compared with 89.1 for 1914 and 84.9 for 1910-1914, and suicide, the rates being respectively 16.8, 15. and 15.5. Births have increased from 23.4 for 1914 and 1910-1914

to 24. Deaths from all causes have decreased from 16.1 the average 1910-1914 and 15.7 for 1914 to 15.3.

---

**The American Medicine Gold Medal** awarded annually for most conspicuous services in medicine and surgery has been awarded this year to Surgeon-General Rupert Blue of the U. S. Public Health Service.

---

**Obstetric Clinics** have been started by the District Nursing Association of Buffalo, at 43 Front avenue and 1553 Broadway, under the supervision of Mrs. Annie L. Hansen, Supt. of the D. N. A. and of Drs. Francis C. Goldsborough, Elmer Clarke and Lorrimer. Especial attention will be given to ante-natal care and, after delivery, the ''Baby Welfare Nurse'' will attend to the children.

---

**The Emergency Hospital of Buffalo** graduated, September 8, Misses Mabel Decker, Leola Phillips, Ellen Gallagher and Angeline Neurohr. Dr. Francis M. Carr presented the diplomas.

---

**Infantile Paralysis at Erie.** 6 cases occurred in the first seven months of this year, 44 between August 1 and September 8. All children under 12 will be excluded from places of amusement and if the epidemic increases, the schools will be closed. The State Health authorities are investigating and it is to be hoped that definite conclusions will be reached regarding the infectious element. The epidemic was undoubtedly furthered by the flood. By the way, the efficiency of the Pennsylvania State Constabulary was again demonstrated during the flood. This body of men, partaking of the nature of police and of regular soldiers obviates, to a large degree, the economic and sociologic disadvantages of calling on the national guard, is available without delay and in its active and past members affords a nucleus of national defense. We hope that New York will adopt such an arm of state service.

---

**Population of New York State.** The 1915 state census shows a population of 9,773,817; Greater N. Y. City having 5,066,222; the rest of the state 4,707,595. The increase for five years is, respectively, 960,864 (11% nearly), 300,661 (6½% nearly), 660,203 (16% plus). Manhattan showed a decrease of 187,481, due largely of course to the lack of vacant space for building and the encroachment of business on residence districts. It should be remembered that for the last year, immigration has been almost totally suspended, if not actually more than balanced by emigration and that, in the past, a disproportionate number of immigrants have remained in N. Y. City. On the whole, the normal increase of population for the state has occurred and mainly for the smaller communities.

---

**Educational Statistics.** In 1914, 216,493 students were regis tered in 567 colleges, and professional schools in the U. S. Of these, there were 139,373 men and 77,120 women, increases over 1913 being 14,262 total, 10,729 men, 3,533 women. The increase of men in a period of financial depression is surprising and it will be noted that the increase of women was disproportionately low—possibly because, having successfully striven for higher education, women are now more interested in getting the franchise. About 6.5% of the total population are included in the four-year age period covered by the college or professional school course and about 2.25% of the total population were actually taking such courses. For males, the respective percentages are about 6.5% and nearly 3%. It occurs to us that a rather serious economic problem is here presented.

---

**''New'' Cure for Leprosy.** It is reported that 23 lepers have been discharged cured from the Culion colony, Phillipines, after treatment by Dr. Mercado, a native physician, with chaulmoogra oil.

---

**Examinations for Assistant Surgeon, U. S. Public Health Service,** will be held at various places, November 1. Application should be made to the Surgeon General at Washington, promptly.

---

**Vaccinations,** according to the N. Y. State law, may be made only by licensed physicians and must be reported on cards issued by the State Health Department.

---

**The University of Buffalo** opened its 70th session, September 20. Addresses were made to the assembled students by Rabbi Copold and Dr. T. H. McKee, the new Dean of the Medical Department. 42 freshman medical students are registered.

---

**Extermination of Typhoid.** The U. S. Public Health Service estimates that 300,000 persons will have been immunized in 1915, as compared with 100,000 last year. In N. Y. State, the deaths for the first half of 1915 were only 5: 100,000 population. In our opinion, there are three principal dangers: gross neglect by cities and villages of well known means of controlling typhoid, under present conditions, almost inevitable neglect of precautions on farms and at summer resorts, and carriers. While vaccination is not claimed to be absolute protection nor to be permanent, its efficiency is very great and it will undoubtedly reduce the incidence. As even a single case may infect many others, such reduction progresses in a geometric series. Moreover, if vaccination can be made universal and can be improved in technic, it will have the inestimable value of rendering unnecessary expensive engineering works aimed at water supplies. In Paris, typhoid has been for several years, almost as rare as diabetes and leucocythaemia and such cases as are seen in the hospitals are almost entirely from outside the city proper.

---

**Abortion Following Rape** is not to be legalized in France. After much discussion, pro and contra, this is the almost unanimous verdict of the medical profession and has undoubtedly had its influence on the civil authorities. The offspring of German soldiers will be cared for by the state.

---

**The Knights of Columbus** have requested action by the managers, to establish a national institution, at a cost of $250,-000.

---

**Four Cases of Infantile Paralysis** have been reported in Elmira this month.

